# Total Polyphenol Content in Food Samples and Nutraceuticals: Antioxidant Indices versus High Performance Liquid Chromatography

**DOI:** 10.3390/antiox11020324

**Published:** 2022-02-07

**Authors:** Oscar Vidal-Casanella, Javier Moreno-Merchan, Merce Granados, Oscar Nuñez, Javier Saurina, Sonia Sentellas

**Affiliations:** 1 Department of Chemical Engineering and Analytical Chemistry, University of Barcelona, Martí i Franquès 1-11, E-08028 Barcelona, Spain; oscarvidalcasanella@gmail.com (O.V.-C.); javiermorenomerchan712@gmail.com (J.M.-M.); mgranados@ub.edu (M.G.); oscar.nunez@ub.edu (O.N.); sonia.sentellas@ub.edu (S.S.); 2 Research Institute in Food Nutrition and Food Safety, University of Barcelona, Recinte Torribera, Avenue Prat de la Riba 171, Edifici de Recerca (Gaudí), E-08921 Santa Coloma de Gramenet, Spain; 3 Serra Húnter Fellow, Generalitat de Catalunya, Rambla de Catalunya 19-21, E-08007 Barcelona, Spain

**Keywords:** polyphenols, antioxidant capacity, high-performance liquid chromatography, ferric reducing antioxidant power, Folin–Ciocalteu, principal component analysis, partial least squares

## Abstract

Total polyphenol content and antioxidant capacity were estimated in various food and nutraceutical samples, including cranberries, raspberries, artichokes, grapevines, green tea, coffee, turmeric, and other medicinal plant extracts. Samples were analyzed by using two antioxidant assays—ferric reducing antioxidant power (FRAP) and Folin–Ciocalteu (FC)—and a reversed-phase high-performance liquid chromatography (HPLC), with a focus on providing compositional fingerprints dealing with polyphenolic compounds. A preliminary data exploration via principal component analysis (PCA) revealed that HPLC fingerprints were suitable chemical descriptors to classify the analyzed samples according to their nature. Moreover, chromatographic data were correlated with antioxidant data using partial least squares (PLS) regression. Regression models have shown good prediction capacities in estimating the antioxidant activity from chromatographic data, with determination coefficients (R^2^) of 0.971 and 0.983 for FRAP and FC assays, respectively.

## 1. Introduction

Polyphenols are ubiquitously present in plants, as secondary metabolites, with one or more phenolic rings in their structures. Thousands of polyphenols can be found in plant-based products, and most of them can be classified, depending on their structures, as phenolic acids, flavonoids, stilbenes, lignans, or tannins [1,2,3].

One of the most important effects of polyphenols, due to their great antioxidant capacity, is their ability to eliminate toxic products that harm organisms through oxidative reactions. Several studies have proven the protective effects of plants against cancers or cardiovascular diseases; these studies have investigated the role of polyphenols. Experimental research has proven that polyphenols, in addition to preventing diseases, could also impact propagation, even in healing.

The separation and determination of polyphenols are difficult tasks, due to the high number of polyphenolic molecules and the matrix complexities of different food samples. Analytical separation methodologies, such as high-performance liquid chromatography (HPLC) and capillary electrophoresis (CE), coupled with several detection systems, including UV–Vis, fluorescence, and mass spectrometry (MS), have been described. The latter is presently the most powerful system used for the identification of polyphenols.

While HPLC is the most common technique used for separating and quantifying individual polyphenols, there are many spectrophotometric assays used to determine the total polyphenol content and, consequently, the antioxidant capacity [4,5]. These spectrophotometric assays are based on chemical reactions, some involving single electron transfer (SET) processes, such as Folin–Ciocalteu (FC) and ferric reducing antioxidant power (FRAP) methods, while others rely on hydrogen atom transfer (HAT) mechanisms (e.g., oxygen radical absorbance capacity (ORAC)), or a combination of the two types (e.g., 2,2′-azino-bis(3-ethylbenzothiazoline-6-sulfonic) acid (ABTS) and 2,2-diphenyl-1-picrylhydrazyl (DPPH) methods) [6,7,8]. Moreover, antioxidant capacity can also be estimated by electrochemical techniques, especially via differential pulse voltammetry (DPV) [1,5].

Focusing on the redox methods evaluated in this work—FC is based on the reduction of Mo (VI) to Mo (V), yielding a blue product often measured at 765 nm. As a drawback, other reducing agents apart from polyphenols—such as ascorbic acid, some sugars, and amino acids—can interfere with the determination, so the content of phenolic compounds may be overestimated [9]. In a similar way, FRAP relies on the reduction of Fe(III) to Fe(II) by the action of the antioxidants. In the presence of 2,4,6-tripyridyl-S-triazine (TPTZ), Fe(II) forms a colored complex that absorbs at 595 nm. Although the assay is simple, the absorption slowly increases over the reaction time and several hours may be required to reach a steady state for some polyphenols (caffeic acid, tannic acid, ferulic acid, ascorbic acid, and quercetin) [10,11].

The lack of solid equivalences between the different indices is a major shortcoming of the spectrophotometric assay, regarding the estimation of the total antioxidant capacity. These discrepancies may be attributed to differences in the mechanisms of the reactions involved, and in sensitivity of each compound towards the index. Furthermore, the great complexities of the food matrices result in potential sources of matrix effects and other chemical interferences [1]. In this regard, chromatographic methods could be of great interest to estimate both the overall and the individual phenolic content of the samples, to try to establish correlations with the antioxidant features. For example, Alén-Ruiz et al. evaluated the influence of major polyphenols on the antioxidant activities of twenty-two Ribeiro red wines made from two different grape varieties. In the study, the correlations between antioxidant activity measured by DPPH and the levels of the different polyphenols obtained by HPLC were poor. This finding was attributed to the strong contribution to the antioxidant activity of wines of the polymerized polyphenols, a fraction of which was not detected by HPLC [12]. Similar results were obtained by Šeruga et al., who determined the polyphenolic content in Croatian wines by reverse-phase HPLC and the antioxidant activity by the FC method. The values obtained by HPLC were lower than those obtained by the FC method, because some compounds, such as proanthocyanidins and various oligomeric phenolics, were likely undetected by the chromatographic method. However, a very good correlation was obtained between the results measured by HPLC and by the FC method, expressed as mg gallic acid equivalents (GAE) [4]. Alonso et al. studied the antioxidant activity of wine by-products and its correlation with specific polyphenolic content [13]. No correlation was found between the polyphenols studied and the antioxidant activity of the different samples. This finding suggests that antioxidant activity is related to the total polyphenolic content, despite some individual polyphenolic compounds contributing more than others [13]. Studies performed to correlate different antioxidant assays, including the FC method, and HPLC determinations of polyphenol cocoa content of Serbian chocolates, showed very strong correlations between the antioxidant activity obtained from all tested assays and the polyphenolic content determined by HPLC [14]. Hawryl et al. studied the relationships between the chromatographic data and total polyphenolic content (FC method) of different basil varieties using the PLS technique. The high value of the determination coefficient (R^2^ = 0.9884) revealed a strong correlation between the content of the phenolic compounds estimated by liquid chromatography and the total phenolic content obtained spectrophotometrically [15]. It can be deduced from the referred studies that the correlations among chromatographic and antioxidant data may depend on the experimental circumstances as well as on the nature of the sample, suggesting that additional research will be needed to draw more solid conclusions.

In this research, we compared the total polyphenolic content estimated from a global chromatographic area at 280 nm with the antioxidant capacity of a wide variety of functional foods and nutraceuticals. Samples were analyzed by HPLC–UV and two spectrophotometric indices (FC and FRAP) to evaluate the antioxidant power. A tentative identification of the main individual contributors to the antioxidant capacity was carried out; we also used HPLC coupled to mass spectrometry (HPLC–MS). The resulting data were characterized by PCA and other chemometric methods to find out sample patterns related to compositional features. Subsequently, HPLC fingerprints were correlated with antioxidant indices by partial least square (PLS) regression.

## 2. Materials and Methods

### 2.1. Reagents and Solutions

The chemicals used in the extraction process, the spectrophotometric assays, and the HPLC method, were methanol and acetonitrile (99.9%, UHPLC Supergradient, PanReac, Barcelona, Spain), formic acid (≥95%, Sigma-Aldrich, St. Louis, MO, USA), hydrochloric acid (37%, PanReac, Barcelona, Spain), Fe (III) chloride and sodium carbonate (Merck, Darmstadt, Germany), FC reagent (PanReac, Barcelona, Spain), and 2,4,6-tripyridyl-S-triazine (TPTZ) (Alfa Aesar, Kandel, Germany). Purified water was generated with an Elix 3 coupled to a Milli-Q system (Bedford, MA, USA). The extraction solvent consisted of MeOH/H_2_O/HCl (70:29:1, *v*:*v*:*v*).

The standards used for spectrophotometric assays were: gallic acid (97.5%) purchased from Sigma Aldrich (St. Louis, MO, USA) and 6-hydroxy-2,5,7,8-tetramethylchroman-2-carboxylic acid (Trolox) purchased from Carbosynth (Berkshire, UK). Solutions of the standards were prepared at 1000 mg L^−1^ (stored in amber vials at 4 °C) using dimethyl sulfoxide (DMSO) as the solvent. The solutions for the calibration were prepared with water to obtain concentrations in the range of 1 to 18 mg L^−1^ gallic acid and 0.2 to 9 mg L^−1^ Trolox, for FC and FRAP, respectively.

### 2.2. Instruments and Apparatus

An Agilent Series 1100 HPLC chromatograph (Agilent, Technologies, Palo Alto, CA, USA) was used, equipped with a binary pump (G1312A), an autosampler (G1379A), a degasser system (G1379A), diode array (DAD, G1315B), and fluorescence (FLD, G1321A) detectors. The separation was carried out with a Kinetex C18 (150 mm length × 4.6 mm I.D, 2.6 µm particle size).

The LC–MS system used for the tentative identification of polyphenols was an Agilent 1100 Series liquid chromatograph coupled to an Applied Biosystems 4000 QTRAP hybrid triple quadrupole/linear ion trap mass spectrometer (AB SCIEX, Framingham, MA, USA).

A Lambda 19 double-beam UV–VIS–NIR spectrophotometer (Perkin Elmer, Waltham, MA, USA) was used for the spectrophotometric assays. Measurements were performed with 10-mm path length cells (QS quartz glass, Hellma, Müllheim, Germany). The absorbance was recorded at 765 nm for the FC assay and at 595 nm for the FRAP assay.

Complementary laboratory equipment comprised an ultrasonic bath (Branson 5510, Danbury, CT, USA) and the Labofuge 400 centrifuge (Heraeus, Hanau, Germany).

### 2.3. Samples

A total of 53 samples from nutraceuticals, foods, and beverages were purchased from supermarkets and herbalist shops in Barcelona (Spain) and Gdansk (Poland). Six types of nutraceuticals (cranberry, cranberry with others, raspberry, black grape (seeds), black grape (peel) with others and grapevine, and artichoke) under the pharmaceutical form of gelatin capsules, 10 types of food products (turmeric, curry, pepper, chocolate, coffee, tea, fruit juice, wine, beer, and sparkling wine) were analyzed. Table S1 summarizes the characteristics of the samples.

### 2.4. Sample Treatment

Two different procedures were followed, depending on the type of sample. Liquid samples (juice, wine, beer, and sparkling wine) were filtered with a syringe through a nylon membrane of a 0.45 µm pore size (20 mm diameter, Macherey-Nagel, Düren, Germany). Filtrates were directly analyzed by HPLC or spectrophotometrically (antioxidant indices) and data were expressed in milligrams of gallic acid or Trolox per liter of sample.

The extraction of polyphenols from solid samples relied on a solvent extraction procedure with an acidified methanolic solution, as described by Vidal-Casanella et al. [16]. Briefly, 0.2 g of sample were mixed with 10 mL of MeOH/H_2_O/HCl (70:29:1, *v*:*v*:*v*) in 15-mL conical tubes. Analytes were recovered by sonication for 30 min at room temperature. Afterwards, the extracts were centrifuged for 15 min at 3200 g and filtered through nylon membranes of a 0.45 µm pore size. Extractions were carried out in triplicate. As above, the resulting solutions were analyzed by HPLC and antioxidant index methods, and concentrations were expressed in milligrams of gallic acid or Trolox per kilogram of sample.

### 2.5. Spectrophotometric Indices

Folin–Ciocalteu (FC): 250 µL of FC reagent and 1 mL of water in an amber vial. After 8 min, 113 µL of a sodium carbonate aqueous solution 7.5% (*w*:*v*) and the appropriate volume of the sample/standard in the calibration range (e.g., 1 to 18 mg L^−1^ gallic acid) were added. The reaction was developed for 2 h. Finally, water was added (up to 5 mL); after 2 h, the absorbance was measured at 765 nm using reagent blank as the reference. The antioxidant capacity was expressed as a gallic acid equivalent. Determinations were carried out in triplicate.

FRAP assay: the FRAP reagent was prepared by mixing 20 mmol L^−1^ FeCl_3_, 10 mmol L^−1^ TPTZ (with 50 mmol L^−1^ HCl), and 50 mmol L^−1^ formic acid aqueous solution in the ratio of (1:2:10, *v*:*v*:*v*). The assay was performed by mixing 300 µL of the FRAP reagent with the appropriate volume of the sample/standard in the calibration range (e.g., 0.2 to 9 mg L^−1^ Trolox). Then, water was added to obtain a final volume of 2.5 mL; after 5 min, the absorbance was measured at 595 nm using reagent blank as the reference. The antioxidant capacity was expressed as a Trolox equivalent. Determinations were carried out in triplicate.

### 2.6. Chromatographic Method

To obtain chromatographic fingerprints of the representative polyphenolic compounds in the samples, an analysis via HPLC–UV/FLD was developed. The compounds were separated by RP mode in a Kinetex C18 (150 mm length × 4.6 mm I.D, 2.6 µm particle size) from Phenomenex (Torrance, CA, USA) using 0.1% (*v*/*v*) HCOOH and ACN as the components of the mobile phase. The elution gradient was as follows: 20% to 40% ACN, from 0 to 12 min (linear increase); 40% to 60% ACN, from 12 to 22 min (linear increase); 60% to 20% ACN, from 22 to 22.1 min (linear decrease). The column was conditioned with 20% ACN for 5 min before the next run. The volume of injection was 5.0 μL and the flow rate was 1 mL min^−1^. The chromatograms were recorded by UV at 280 nm and by FLD at 280 and 330 nm as the excitation and emission wavelengths, respectively.

A quality control (QC) prepared by mixing 25 µL of each sample extract was analyzed every 10 sample injections to assess both the repeatability of the chromatographic data and the quality of the PCA model.

For identification purposes, the LC–MS/MS (MRM mode) chromatogram of samples were compared to that of polyphenol standards. Chromatographic conditions were as above. Regarding mass spectrometry parameters, polyphenols were detected in negative mode. The ion spray voltage was set at −2500 V and the source temperature was 700 °C. Nitrogen was used as a nebulizer and auxiliary gas and was set at 20, 50, and 50 arbitrary units for the curtain gas, the ion source gas 1, and the ion source gas 2, respectively. Declustering potential (DP), collision energy (CE), collision exit cell potential (CXP), and ion transitions pairs were optimized for each available standard (Table S2).

### 2.7. Data Analysis

A PLS-Toolbox (Eigenvector Research, Manson, WA, USA) with MATLAB was used for the exploratory and classification studies of the 53 samples from nutraceuticals, foods, and beverages.

Principal component analysis (PCA) was used for sample characterization using the chromatographic fingerprints of samples. For the HPLC–UV analysis, the X-matrix consisted of 177 samples × 3721 absorbance data, including 53 samples analyzed in triplicate plus 18 QCs, and the time range was 1.15 to 25.95 min. The chromatographic segments corresponding to the front and the cleaning steps were removed since no relevant information dealing with phenolic/antioxidant species was present. For the HPLC–FLD analysis, the X-matrix consisted of 177 samples × 3330 fluorescence intensities within the working time window. The chromatographic segments corresponding to the front and the cleaning steps were removed from the analysis, since no relevant data dealing with phenolic/antioxidant species were present. The plots of scores showing the distributions of the samples on the first and second principal components (PCs) were used to differentiate the samples according to their matrix. The loading plot allowed to identify the most discriminant polyphenolic compounds.

Partial least squares (PLS) regression was applied to predict the response in the antioxidant indices (Y-matrix) as a function of the chromatographic data obtained (X-matrix). Theoretical information about the chemometric methods can be found elsewhere [17,18,19].

## 3. Results and Discussion

### 3.1. HPLC and MS Analysis

**Figure 1:**
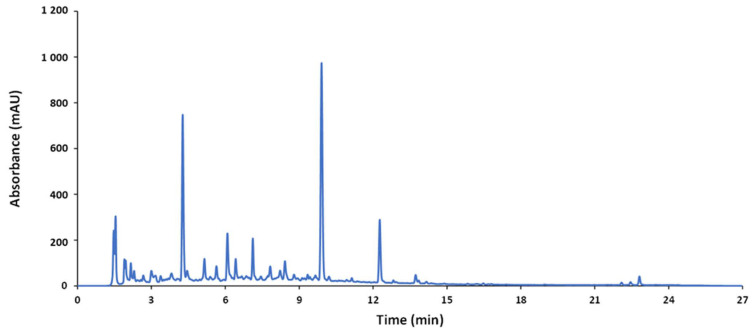
Chromatogram of the QC recorded by UV at 280 nm.

A reverse-phase HPLC method was used to analyze the wide variety of samples (nutraceuticals, foods, and beverages). The optimization was focused on obtaining compositional profiles of the samples under study---as rich as possible at a minimum running time. For this purpose, the QC sample was used as a representative average sample. A suitable elution gradient based on previous studies is given in the experimental section. The chromatogram of the QC sample obtained under these conditions is depicted in Figure 1. Since sample extracts consisted of complex mixtures of a wide range of compounds with different spectroscopic features and polarity, a gradient profile increasing the ACN percentage from 20% to 60% in 22 min (total running time, 27 min, including separation, cleaning, and stabilization steps) was applied to achieve a reasonable separation of the compounds.

Representative chromatograms of each sample type recorded by UV and FLD are depicted in Figure S1 (see Supplementary Materials). As can be seen, noticeable differences depending on the nature of the sample can be found in both types of fingerprints. This finding suggests that given products could be discriminated from each other based on the differences in composition. In the case of UV chromatograms recorded at 280 nm, they mainly contained information dealing with phenolic acids and some flavonoid families, so that the overall areas could be reasonable indices of the global phenolic content closely related to the global antioxidant activity [20]. In contrast, FLD fingerprints were more specific of flavanol and flavanone species, while the contributions of other phenolic compounds, such as hydroxybenzoic and hydroxycinnamic acids, were negligible [21].

A tentative identification of some major phenolic components in each type of sample relied on HPLC–MS (see Table S3). A targeted study using standards revealed that, in the case of chocolate—epicatechin, catechin, gallic acid, procyanidin C1, and procyanidin B1 were major phytochemicals. The most significant flavonoids in tea were epigallocatechin, quercetin, rutin, myricetin, and hesperidin. Moreover, caffeic and related compounds, such as chlorogenic acid, caftaric, and coumaric acids, and 3,4-dihydroxybenzoic acid and 4-hydroxybenzoic acid, were also important. Coffee extracts were rich in hydroxycinnamic acids, such as ferulic and caffeic acid, and their derivatives, especially chlorogenic acids. In the case of turmeric and curry, the most significative compounds were curcuminoids, including curcumin, demethoxycurcumin, and bisdemethoxycurcumin, which is of great interest because of their anti-inflammatory and antineoplastic activities; caffeic acid, coumaric acid, ferulic acid, 4-hydroxybenzoic acid, vanillic acid, and vanillin were also abundant. Cranberry-based extracts were rich in flavanols—epicatechin, procyanidin C1, and procyanidin B1—among which, procyanidin A2 stands out, due to its antibacterial activity. For grape and wine products—caffeic, caftaric, and coutaric acid were the major species, with gallic acid, ethyl gallate, epicatechin, and chlorogenic acid also occurring at high levels. Raspberry showed some components similar to other berries, comprising flavanols, such as epigallocatechin, epicatechin, and catechin, flavonols, such as quercetin, and phenolic acids, such as caffeic and gallic acids. The main phenolic compounds in artichoke extract were hydroxycinnamic acids (e.g., ferulic, coumaric, and caffeic acids), and multiple hydroxybenzoic acids (e.g., gallic, 3,4-dihydroxybenzoic, 4-hydroxybenzoic, vanillic acid and 2,5-dihydroxybenzoic acids). Finally, pepper samples contained some flavonoids (e.g., quercetin) and phenolic acids (e.g., caffeic and 3,4-dihydroxybenzoic acids). These results agree with previous studies that focused on the characterization of each particular matrix [16,20,21,22,23,24,25,26].

### 3.2. Sample Characterization by PCA

**Figure 2:**
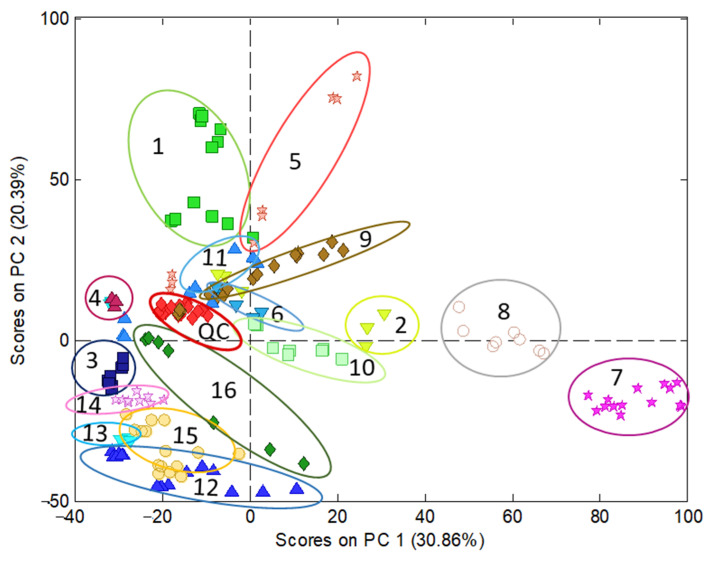
Characterization of nutraceuticals, foods, and beverages by PCA using the chromatographic fingerprints by UV (280 nm) in the time range 1.15 to 25.95 min as the data. Scatter plot of scores of PC1 vs. PC2. Classes identification: 1 = cranberry extract; 2 = cranberry (with other plants) extract; 3 = raspberry extract; 4 = black grape (seeds) extract; 5 = black grape (peel and grapevine) extract; 6 = artichoke extract; 7 = turmeric extract; 8 = curry extract; 9 = coffee extract; 10 = pepper extract; 11 = tea extract; 12 = juice; 13 = wine; 14 = beer; 15 = sparkling wine; 16 = chocolate extract.

Regarding polyphenolic fingerprinting, the data under study consisted of sample chromatograms recorded by UV at 280 nm, in the working range of 1.15 to 25.95 min, in which the most significant components were eluted, especially those corresponding to phenolic acids and flavonoids. Moreover, to obtain additional information for the compositional profiles, chromatograms recorded by FLD at 280 and 330 nm as the excitation and emission wavelengths, respectively, in the working range of 1.22 to 25.19 min, were also examined. The data pretreatment consisted of a correction of the baseline, standardization by the sample mass, normalization, and autoscaling. Then, chemometric analyses by the PCA of UV and FLD data were conducted. The UV model (Figure 2) showed that QCs appear clustered, nearly in the middle of the model; therefore, this indicates a good reproducibility of chromatographic data and suitability of the model built. PC1 mainly discriminated among turmeric-based (on the right side) and other samples (on the left). According to the information provided by PCA loadings, curry, especially turmeric samples, were highly differentiated from the others by the content of curcuminoids, which presented high retention times (22–23 min) because of their lower polarity compared with other polyphenols. Conversely, those samples on the left side displayed more polar molecules, such as hydroxycinnamic acids regarding wines and some berries. PC2 discriminated samples as a function of the overall phenolic content, with the richest extracts located in the upper part of the graph (e.g., cranberry and grape-based products) while the less concentrated ones were at the bottom (juices and other beverages).

Figure S2 shows the characterization of the samples by PCA from FLD data recorded at 280 and 330 nm as the excitation and emission wavelengths. The principal difference between the UV and FLD model is that, in the first, the cranberry samples are more dispersed than in the FLD model and mixed with samples made of black grape (peel) with others and grapevine. Moreover, the variance explained with two PCs by the UV model is higher than that captured by the FLD counterpart—51.25% and 45.04%, respectively. Eventually, the results obtained by UV were considered better than by FLD. This finding was attributed to the higher richness of the UV fingerprints, containing information from a wide range of phenolic acid and flavonoid families, while FLD data were more limited to flavanols and flavanones [21].

As a conclusion, PCA models revealed interesting information on the compositions of the samples from both qualitative and quantitative points of view; they were mainly distributed depending on the polarities of their phenolic components as well as their concentrations.

### 3.3. Determination of the Antioxidant Capacity of Different Sample Classes by Spectrophotometric Indices

The 53 samples from nutraceuticals, foods, and beverages were also analyzed via FC and FRAP methods to assess the polyphenol content expressed as the antioxidant capacity (g standard per Kg or L of sample). Under the current circumstances, the FC method was linear in the range 1 to 18 mg L^−1^ gallic acid (determination coefficient, R^2^ = 0.994) and the FRAP method was linear between 0.2 and 9 mg L^−1^ Trolox (R^2^ = 0.998). Figure S3 shows a comparison of the antioxidant capacities of different sample classes by FC and FRAP indices.

**Figure 3:**
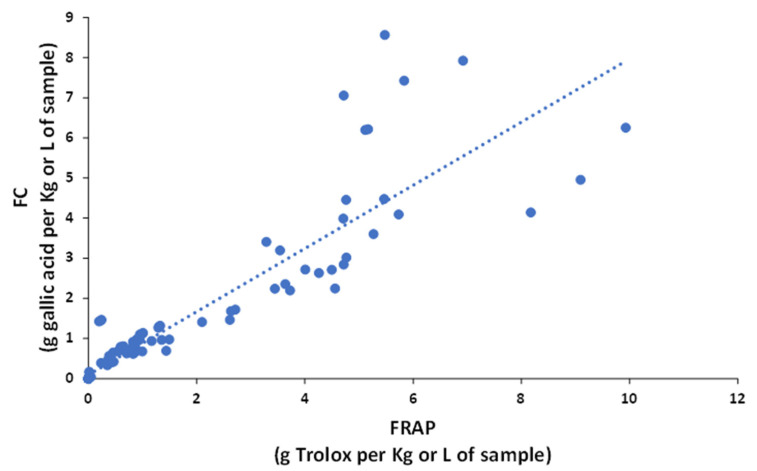
Correlation of the antioxidant capacity between Folin–Ciocalteu (FC) and FRAP.

Based on these indices, samples with higher antioxidant activities were tea and berry extracts (e.g., from cranberry, black grape, and raspberry), while beverages, such as beer, peach juice, and sparkling wine showed lower activities. The comparison of results from the different indices reveal that the antioxidant power of each sample type depended on the FC or FRAP method because the sensitivities towards each type of polyphenol were different [12]. For example, even though FC and FRAP reactions follow the same antioxidant HAT mechanism and the redox potentials of both systems are similar, the FC index is more sensitive in cranberry and raspberry samples, and the FRAP index in artichoke, coffee, and tea samples. These discrepancies can be easily visualized in the plot of FC versus FRAP data (Figure 3), in which, despite the correlation, was statistically significant (R^2^ = 0.8259); some samples differed from the general trend.

The antioxidant power by FC and FRAP were also compared with the total polyphenolic content estimated by HPLC–UV. This parameter, relying on the total area of chromatographic peaks detected at 280 nm, was found to be an excellent descriptor of the overall phenolic concentration, so we expected a reasonable correlation with the antioxidant capacity of the samples. Figure S4 shows the correlation studies of FC versus HPLC and FRAP versus HPLC–UV data. In the two cases, correlations were statistically significant (*p* < 0.05), with determination coefficients of 0.8595 and 0. 7755, respectively. These findings suggested that the vast majority of compounds detected at 280 nm displayed phenolic moieties in their structures that, eventually, provided antioxidant capacity. In this case, the redox processes involved were mainly related to the oxidation of phenolic groups to quinones.

### 3.4. Correlation between HPLC Fingerprints and Spectrophotometric Indices by PLS

PLS was applied to investigate the possibility of estimating the antioxidant capacity from the chromatographic data. The PLS analysis was conducted with HPLC–UV fingerprints at 280 nm as the X-matrix and the antioxidant capacities of the samples for FC or FRAP indices in the Y-matrix. As indicated above, UV chromatograms at 280 nm were taken in the working range of 1.15 to 25.95 min, in which those relevant compounds were eluted, while avoiding interferences from the chromatographic front and cleaning range. In any case, a PCA model was built to remove the outliers from the exploration of the graphics of Q residuals vs. Hotelling’s T^2^.

**Figure 4:**
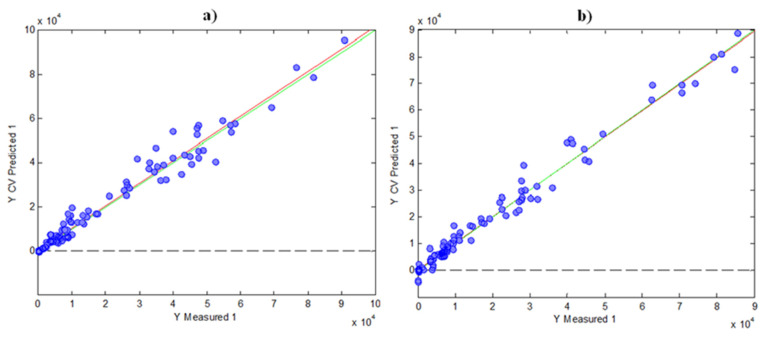
Graphics of Y measured vs. Y CV predicted for PLS models by using the data of indices as matrix Y: (**a**) FC; (**b**) FRAP.

For the regression model using FC results, six LVs were found optimal to carry out the calibration, as was deduced by cross validation (CV) under a Venetian blind approach. In these circumstances, the variance explained was 77.53% for the X-block and 99.63% for the Y-block. Figure 4a shows the scatter plot of FC indices measured vs. FC indices predicted by cross-validation using PLS. As can be seen, the prediction was accurate, with a R^2^ of CV of 0.983.

For the FRAP prediction, a PLS model using HPLC fingerprints and FRAP results was built in a similar way, as in the case of FC. Four LVs were found optimal in this case, with a variance explained of 73.50% for the X-block and 98.16% for the Y-block. Figure 4b shows the measured FRAP values vs. CV-predicted FRAP values, in which the R^2^ of CV was 0.971. Again, these results indicate a good prediction with the two models, having a high correlation with the HPLC data.

Figure S5 shows the regression vector for the prediction of antioxidant indices from the chromatographic fingerprints by the FC and FRAP models. As observed, the differences between the two indices depend principally on the sensitivities to different polyphenolic compounds, since the same zones on the chromatograms positively affect both indices.

## 4. Conclusions

Chromatographic fingerprints recorded by HPLC–UV and HPLC–FLD demonstrated great value in characterizing samples according to polyphenolic components. By comparing the performance of the two approaches, HPLC–UV at 280 nm seemed to be more effective than HPLC–FLD at 280/330 nm (excitation/emission wavelengths) when dealing with the discrimination of food and nutraceutical samples. Hence, exploratory studies by PCA using HPLC–UV fingerprints showed excellent sample clustering according to their compositional fingerprints.

On the other hand, the analysis of the samples by FC and FRAP spectrophotometric indices exhibited some differences in the antioxidant capacity, depending on the type of sample. This finding was attributed to the different sensitivities of components (or samples) toward each index. Despite these differences, similar overall conclusions were drawn from both FC and FRAP results, showing that, regardless of the index, the sample extracts with higher antioxidant capacities were berries (cranberry and raspberry), black grapes, and tea.

The potential relationship between chromatographic fingerprints and antioxidant capacity was also investigated. In general, it was found that the overall chromatographic areas of both UV and FLD profiles positively correlated with the FC or FRAP data. As a result, the antioxidant capacity of a sample, in terms of either FC or FRAP equivalents, was estimated by PLS using the chromatographic profile as the source of information. In the two cases, a good predictive performance was obtained, which indicated that chromatograms could be successfully used to estimate the antioxidant capacities of these food or nutraceutical extracts.

## Data Availability

Data are contained within the article and Supplementary Material.

## Supplementary Materials

The following supporting information can be downloaded at: https://www.mdpi.com/article/10.3390/antiox11020324/s1. Table S1. List of samples analyzed and their characteristics; Table S2. MRM transitions for the detection of polyphenols by LC–ESI–MS/MS; Table S3. Principal polyphenols identified in the different types of samples; Figure S1. Chromatographic profiles of a representative sample of each type: (a) UV (λ = 280 nm); (b) FLD (λex = 280 nm, λem = 330 nm); Figure S2. Characterization of nutraceuticals, foods and beverages by PCA using the chromatographic fingerprints by FLD at 280 and 330 nm as the excitation and emission wavelengths in the time range 1.22 to 25.19 min as the data. Scatter plot of scores of PC1 vs. PC2. Classes identification: 1 = cranberry; 2 = cranberry with others; 3 = raspberry; 4 = black grape (seeds); 5 = black grape (peel) with others and grapevine; 6 = artichoke; 7 = turmeric; 8 = curry; 9 = coffee; 10 = pepper; 11 = tea; 12 = juice; 13 = wine; 14 = beer; 15 = sparkling wine; 16 = chocolate; Figure S3. Determination of antioxidant capacity by FC and FRAP indices as acid gallic or Trolox equivalents, respectively, on different samples classes. Error bars indicate the standard deviation from three independent replicates; Figure S4. Correlation studies: (a) FRAP versus HPLC–UV; (b) FC versus HPLC–UV; Figure S5. Regression vector for the prediction of the antioxidant index vs. variables (chromatographic range): (a) FC; (b) FRAP.

## References

[1] Alcalde B., Granados M., Saurina J. (2019). Exploring the Antioxidant Features of Polyphenols by Spectroscopic and Electrochemical Methods. Antioxidants.

[2] Arts I.C.W., Hollman P.C.H. (2005). Polyphenols and Disease Risk in Epidemiologic Studies. Am. J. Clin. Nutr..

[3] Brglez Mojzer E., Knez Hrnčič M., Škerget M., Knez Ž., Bren U. (2016). Polyphenols: Extraction Methods, Antioxidative Action, Bioavailability and Anticarcinogenic Effects. Molecules.

[4] Šeruga M., Novak I., Jakobek L. (2011). Determination of Polyphenols Content and Antioxidant Activity of Some Red Wines by Differential Pulse Voltammetry, HPLC and Spectrophotometric Methods. Food Chem..

[5] Vidal-Casanella O., Nunez O., Granados M., Saurina J., Sentellas S. (2021). Analytical Methods for Exploring Nutraceuticals Based on Phenolic Acids and Polyphenols. App. Sci..

[6] Ebner H., Dienstbach F., Sandritter W. (1967). Hormonelle Beeinflussung des experimentellen Portiocarcinoms. Natl. Libr. Med..

[7] Re R., Pellegrini N., Proteggente A., Pannala A., Yang M., Rice-Evans C. (1999). Antioxidant Activity Applying an Improved ABTS Radical Cation Decolorization Assay. Free Radic. Biol. Med..

[8] Brand-Williams W., Cuvelier M.E., Berset C. (1995). Use of a Free Radical Method to Evaluate Antioxidant Activity. LWT Food Sci. Technol..

[9] Everette J.D., Bryant Q.M., Green A.M., Abbey Y.A., Wangila G.W., Walker R.B. (2010). Thorough Study of Reactivity of Various Compound Classes toward the Folin-Ciocalteu Reagent. J. Agric. Food Chem..

[10] Apak R., Özyürek M., Güçlü K., Çapanoʇlu E. (2016). Antioxidant Activity/Capacity Measurement. 1. Classification, Physicochemical Principles, Mechanisms, and Electron Transfer (ET)-Based Assays. J. Agric. Food Chem..

[11] Ou B., Huang D., Hampsch-Woodill M., Flanagan J.A., Deemer E.K. (2002). Analysis of Antioxidant Activities of Common Vegetables Employing Oxygen Radical Absorbance Capacity (ORAC) and Ferric Reducing Antioxidant Power (FRAP) Assays: A Comparative Study. J. Agric. Food Chem..

[12] Alén-Ruiz F., García-Falcón M.S., Pérez-Lamela M.C., Martínez-Carballo E., Simal-Gándara J. (2009). Influence of Major Polyphenols on Antioxidant Activity in Mencía and Brancellao Red Wines. Food Chem..

[13] Alonso Á.M., Guillén D.A., Barroso C.G., Puertas B., García A. (2002). Determination of Antioxidant Activity of Wine Byproducts and Its Correlation with Polyphenolic Content. J. Agric. Food Chem..

[14] Todorovic V., Redovnikovic I.R., Todorovic Z., Jankovic G., Dodevska M., Sobajic S. (2015). Polyphenols, Methylxanthines, and Antioxidant Capacity of Chocolates Produced in Serbia. J. Food Compos. Anal..

[15] Hawrył A., Hawrył M. (2020). Chromatographic Fingerprinting of Some Basils and the Evaluation of Their Antioxidant Properties with Chemometric Calculations. J. Liq. Chromatogr. Relat. Technol..

[16] Vidal-Casanella O., Arias-Alpizar K., Nuñez O., Saurina J. (2021). Hydrophilic Interaction Liquid Chromatography to Characterize Nutraceuticals and Food Supplements Based on Flavanols and Related Compounds. Separations.

[17] Jolliffe I.T., Cadima J. (2016). Principal Component Analysis: A Review and Recent Developments. Philos. Trans. Ser. A Math. Phys. Eng. Sci..

[18] Kozak M., Scaman C.H. (2008). Unsupervised classification methods in food sciences: Discussion and outlook. J. Sci. Food Agric..

[19] Granato D., Putnik P., Kovacevic D.B., Santos J.S., Calado V., Rocha R.S., Da Cruz A.G., Jarvis B., Rodionova O.Y., Pomerantsev A. (2018). Trends in Chemometrics: Food Authentication, Microbiology, and Effects of Processing. Compr. Rev. Food Sci. Food Saf..

[20] Tapia-Quiros P., Montenegro-Landivar M.F., Reig M., Vecino X., Alvarino T., Cortina J.L., Saurina J., Granados M. (2020). Olive Mill and Winery Wastes as Viable Sources of Bioactive Compounds: A Study on Polyphenols Recovery. Antioxidants.

[21] Bakhytkyzy I., Nunez O., Saurina J. (2018). Determination of flavanols by liquid chromatography with fluorescence detection. Application to the characterization of cranberry-based pharmaceuticals through profiling and fingerprinting approaches. J. Pharm. Biomed. Anal..

[22] Izquierdo-Llopart A., Saurina J. (2020). Liquid Chromatographic Approach for the Discrimination and Classification of Cava Samples Based on the Phenolic Composition Using Chemometric Methods. Beverages.

[23] Vidal-Casanella O., Nunez N., Sentellas S., Nunez O., Saurina J. (2020). Characterization of Turmeric and Curry Samples by Liquid Chromatography with Spectroscopic Detection Based on Polyphenolic and Curcuminoid Contents. Separations.

[24] Barbosa S., Campmajo G., Saurina J., Puignou L., Nunez O. (2020). Determination of Phenolic Compounds in Paprika by Ultrahigh Performance Liquid Chromatography-Tandem Mass Spectrometry: Application to Product Designation of Origin Authentication by Chemometrics. J. Agric. Food Chem..

[25] Barbosa S., Pardo-Mates N., Hidalgo-Serrano M., Saurina J., Puignou L., Nunez O. (2018). Detection and Quantitation of Frauds in the Authentication of Cranberry-Based Extracts by UHPLC-HRMS (Orbitrap) Polyphenolic Profiling and Multivariate Calibration Methods. J. Agric. Food Chem..

[26] Pardo-Mates N., Vera A., Barbosa S., Hidalgo-Serrano M., Nunez O., Saurina J., Hernandez-Cassou S., Puignou L. (2017). Characterization, classification and authentication of fruit-based extracts by means of HPLC-UV chromatographic fingerprints, polyphenolic profiles and chemometric methods. Food Chem..

