# Supplementary material for: Total Polyphenol Content in Food Samples and Nutraceuticals: Antioxidant Indices versus High Performance Liquid Chromatography
Source: Antioxidants (Basel). 2022 Feb 7;11(2):324. doi: 10.3390/antiox11020324 (PMC8868288; doi:10.3390/antiox11020324)
Supplement: Supplementary file 1 [file antioxidants-11-00324-s001.zip › antioxidants-1572929-supple.pdf]

Article

Total Polyphenol Content in Food Samples and Nutraceuticals: Antioxidant Indices versus High Performance Liquid Chromatography

Oscar Vidal-Casanella[1], Javier Moreno-Merchan[1], Merce Granados[1], Oscar Nuñez[1,2], Javier Saurina[1,2,*], Sonia Sentellas[1,2,3]

[1] Department of Chemical Engineering and Analytical Chemistry, University of Barcelona. Martí i Franquès, 1-11, E-08028 Barcelona, Spain.
[2] Research Institute in Food Nutrition and Food Safety, University of Barcelona, Recinte Torribera, Av. Prat de la Riba 171, Edifici de Recerca (Gaudí), E-08921 Santa Coloma de Gramenet, Barcelona, Spain.
[3] Serra Húnter Fellow, Generalitat de Catalunya, Spain.
* xavi.saurina@ub.edu

Table S1. List of samples analyzed and their characteristics.

Sample type	Identification code and composition	
**Cranberry** (Gelatin capsule)	EC1	*Vaccinium macrocarpon*
	EC2	
	EC3	
	C02	
**Cranberry with others** (Gelatin capsule)	C04	*Vaccinium macrocarpon, Urtica urens*
	C05	*Vaccinium macrocarpon, Solidago virgaurea*, vitamin D
**Raspberry** (gragea)	C17	*Rubus idaeus, Mangillera Indica, Camellia sinensis, Paullina cupana, Euterpe olercarcea*, L-carnitine
	C18	*Rubus idaeus, Camellia sinensis*
	C19	*Rubus idaeus, Camellia sinensis*
**Black grape (seeds)** (Gelatin capsule)	C20	*Vitis vinifera* (seeds)
**Black grape (peel) with others and grapevine** (Gelatin capsule)	C21	*Vitis vinifera* (peel), *Allium cepa*, *Polygonum cuspidatum*
	C22	*Vitis vinifera* (peel), *Punica granatum*
	C23	*Vitis vinifera* (leaves extract)
**Artichoke** (Gelatin capsule)	C25	*Cynara scolymus*
	C26	
**Turmeric** (powdered root)	T1	*Curcuma longa* (Alleppey)
	T2	*Curcuma longa* (Erode)
	T3	

	T4	*Curcuma longa* (Madras)
	T5	*Curcuma zedoaria*
**Curry** (commercial species)	CY1	Turmeric, pepper, coriander, cumin, fenugreek, parsley, chili, garlic, fennel
	CY2	Turmeric, pepper, coriander, ginger, cumin, fenugreek, laurel, fennel, mustard
	CY3	Turmeric, pepper, coriander, fennel, cumin, cayenne, garlic, anise
**Coffee** (grounded)	CO1	Coffee *Robusta*
	CO2	Coffee *Arabica*
	CO3	
	CO4	Coffee *Robusta*
	CO5	
	CO6	Coffee *Arabica-Robusta*
**Pepper** (commercial species)	P1	Hot pepper (PDO de la Vera)
	P2	Hot pepper (PDO Murcia)
	P3	Hot Pepper (PDO Mallorca)
**Tea** (commercial product)	Te1	Green tea
	Te2	Red tea with anise
	Te3	White tea with vanilla
**Juice**	J1	Peach
	J2	Pineapple
	J3	tomato
	J4	Apple
	J5	Pear
**Wine**	W1	white wine (PDO Gandesa)
	W2	red wine (PDO Priorat)
**Beer**	B1	Lager
	B2	Lager
	B3	India Pale Ale
**Sparkling wine**	S1	White (grape variety: Macabeu, Xarel·lo and Parellada)
	S2	Rosé (grape variety: Pinord noir and Chardonnay)
	S3	White (grape variety: Macabeu, Xarel·lo and Parellada)
	S4	White (grape variety: Macabeu, Xarel·lo and Parellada)
	S5	Rosé (grape variety: Pinord noir and Chardonnay)
**Chocolate**	CH1	dark (coccoa content, 100%)
	CH2	dark (coccoa content, 99%)
	CH3	dark (coccoa content, 85%)

Table S2. MRM transitions for the detection of polyphenols by LC-ESI-MS/MS.

**Polyphenol**	**Parent ion (*m/z*)**	**Daughter ion (*m/z*)**	**DP (V)**	**CE (V)**	**CXP (V)**
Gallic acid	169.0	124.9	-40	-22	-19
Caffeic acid	178.9	134.8	-45	-20	-1
Quercetin	300.9	150.8	-80	-32	-1
Hesperidin	609.3	301.2	-115	-36	-19
Resveratrol	226.8	143.3	-70	-34	-1
Ferulic acid	192.6	134.3	-30	-22	-1
Vanillic acid	166.9	151.8	-65	-20	-1
Ethyl gallate	197.2	123.8	-60	-34	-3
Ellagic acid	300.8	284.0	-95	-98	-1
Catechin	289.1	109.2	-110	-42	-19
Epicatechin	288.7	124.9	-85	-33	-7
Epigallocatechin	304.9	125.0	-80	-26	-25
p-Coumaric acid	163.0	119.3	-60	-18	-1
Rutin	609.2	299.6	-95	-48	-23
Myricetin	316.9	150.8	-95	-40	-9
Syringic acid	196.9	120.9	-40	-26	-9
Astilbin	449.1	285.0	-100	-32	-1
Trans-Coutaric acid	295.0	162.9	-30	-20	-11
Caftaric acid	311.1	179.0	-50	-22	-11
Diosmin	607.3	299.1	-105	-34	-23
Hesperetin	301.0	286.1	-105	-36	-5
Naringin	579.3	271.0	-140	-36	-5
Naringenin	271.0	150.8	-90	-32	-25
Catechol	109.1	90.9	-25	-26	-5
4-hydroxybenzoic acid	136.8	92.9	-55	-18	-7
Vanillin	150.9	135.9	-50	-14	-31
Chlorogenic acid	352.9	190.6	-60	-20	-17
3-methylcatechol	122.9	108.0	-50	-22	-13
4-ethylcatechol	136.9	122.0	-80	-22	-5
2,5-dihydroxybenzoic acid	152.8	108.0	-50	-18	-15
4-methylcatechol	122.9	108.1	-75	-22	-7
3,4-dihydroxibenzoic acid	152.9	108.9	-70	-20	-3
Procyanidin A2	575.2	285.2	-135	-38	-1
Procyanidin B2	577.1	407.1	-5	-26	-11
Procyanidin C1	865.3	125.1	-180	-86	-7

DP: declustering potential, CE: collision energy, CXP: collision cell exit potential.

Table S3. Principal polyphenols identified in the different types of samples.

Sample type	Main polyphenol(s)	Other minor polyphenols
Cranberry	3,4-dihydroxybenzoic acid; p-coumaric acid; vanillic acid; gallic acid; caffeic acid; ferulic acid; epicatechin; catechin; quercetin; myricetin; chlorogenic acid; procyanidin B1; procyanidin A2; procyanidin C1	4-hydroxybenzoic acid; 2,5-dihydroxybenzoic acid; vanillin; syringic acid; epigallocatechin
Raspberry	gallic acid; caffeic acid; epicatechin; catechin; quercetin; epigallocatechin	3,4-dihydroxybenzoic acid; vanillic acid; ferulic acid; chlorogenic acid; rutin; hesperitin
Grape	4-hydroxybenzoic acid; 2,5-dihydroxybenzoic acid; 3,4-dihydroxybenzoic acid; p-coumaric acid; vanillic acid; gallic acid; caffeic acid; ferulic acid; resveratrol; epicatechin; catechin; quercetin; caftaric acid; coutaric acid; stilbin; diosmin; rutin	catechol; 3-methylcatechol; 4-methylcatechol; vanillin; syringic acid; ethyl gallate; naringenin; myricetin; hesperitin
Grapevine	4-hydroxybenzoic acid; 3,4-dihydroxybenzoic acid; p-coumaric acid; vanillic acid; gallic acid; caffeic acid; ferulic acid; caftaric acid; coutaric acid; quercetin; rutin; hesperitin	catechol; 3-methylcatechol; 4-methylcatechol; 2,5-dihydroxybenzoic acid; syringic acid; resveratrol; epicatechin; catechin; epigallocatechin myricetin; stilbin; diosmin
Turmeric	4-hydroxybenzoic acid; vanillin; p-coumaric acid; vanillic acid; caffeic acid; ferulic acid; curcumin; demethoxycurcumin; bisdemethoxycurcumin	3-methylcatechol; 4-methylcatechol; 3,4-dihydroxybenzoic acid; gallic acid;
Coffee	3,4-dihydroxybenzoic acid; caffeic acid; ferulic acid; chlorogenic acid	catechol; ethyl catechol; coumaric acid; vanillic acid; gallic acid
Pepper	4-hydroxybenzoic acid; 3,4-dihydroxybenzoic acid; p-coumaric acid; caffeic acid; ferulic acid; quercetin; chlorogenic acid; rutin; hesperitin	catechol; ethyl catechol; 3-methylcatechol; 4-methylcatechol; 2,5-dihydroxybenzoic acid; vanillic acid; gallic acid; syringic acid, stilbin
Tea	4-hydroxybenzoic acid; 3,4-dihydroxybenzoic acid; p-coumaric acid; gallic acid; caffeic acid; ferulic acid; epicatechin; catechin; epigallocatechin; quercetin; myricetin; chlorogenic acid; rutin; hesperitin	2,5-dihydroxybenzoic acid; vanillic acid; naringenin; naringin
Beer	caffeic acid	4-hydroxybenzoic acid; 3,4-dihydroxybenzoic acid; p-coumaric acid;<br>vanillic acid; ferulic acid; ethyl gallate; naringin

Red wine	4-hydroxybenzoic acid; 3,4-dihydroxybenzoic acid; p-coumaric acid; vanillic acid; gallic acid; caffeic acid; ferulic acid; ethyl gallate; resveratrol; epicatechin; catechin; coutaric acid; quercetin; myricetin; stilbin	3-methylcatechol; 4-methylcatechol; 2,5-dihydroxybenzoic acid; syringic acid; epigallocatechin;
Chocolate	3,4-dihydroxybenzoic acid; caffeic acid; epicatechin; procyanidin B2	4-hydroxybenzoic acid; p-coumaric acid; vanillic acid; gallic acid; catechin; quercetin; stilbin
Artichoke	3,4-dihydroxybenzoic acid; p-coumaric acid; vanillic acid; gallic acid; caffeic acid; ferulic acid; chlorogenic acid; stilbin	4-hydroxybenzoic acid; 2,5-dihydroxybenzoic acid; syringic acid; caftaric acid; diosmin

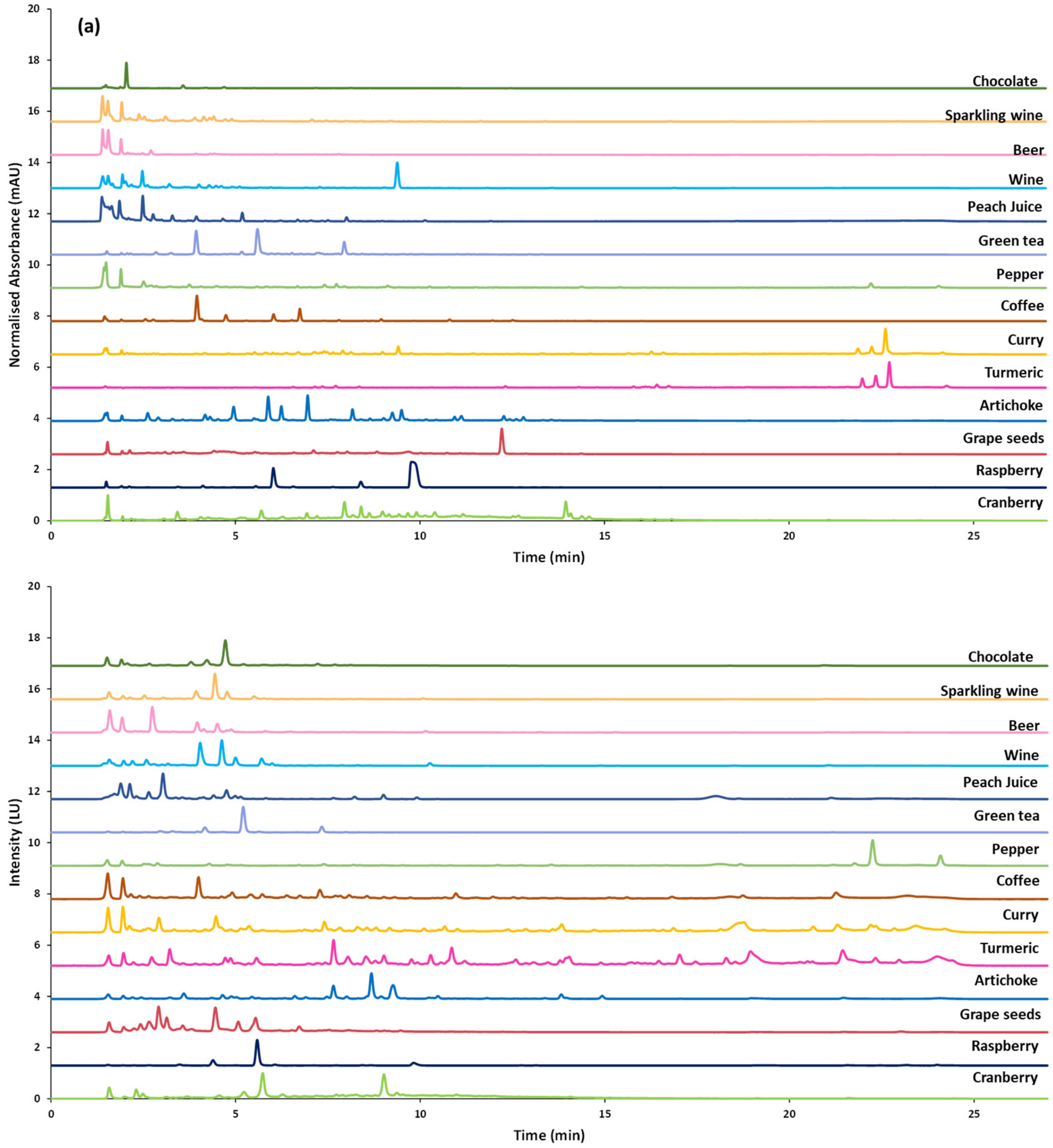

Figure S1. Chromatographic profiles of a representative sample of each type: a) UV (λ=280 nm); b) FLD (λ_{ex}=280 nm, λ_{em}=330 nm).

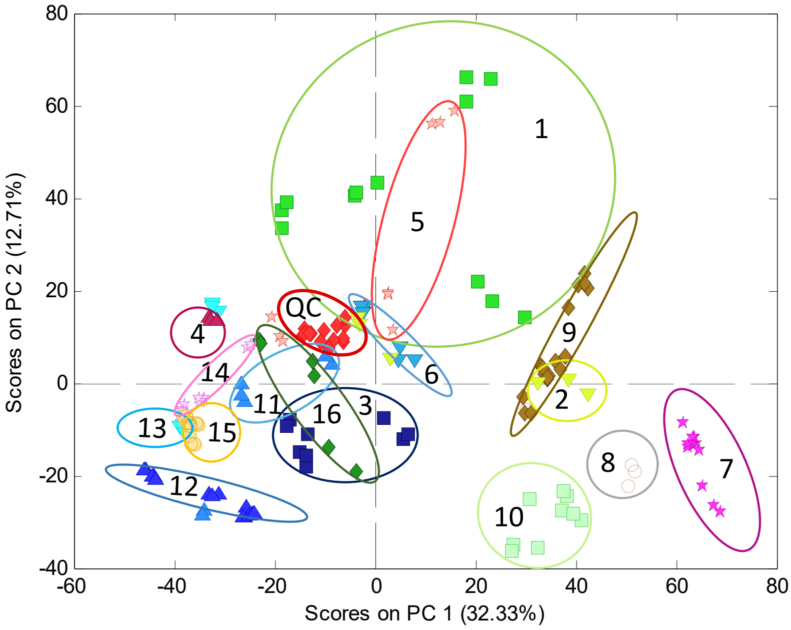

Figure S2. Characterization of nutraceuticals, foods and beverages by PCA using the chromatographic fingerprints by FLD at 280 and 330 nm as the excitation and emission wavelengths in the time range 1.22 to 25.19 min as the data. Scatter plot of scores of PC1 vs PC2. Classes identification: 1 = Cranberry; 2 = Cranberry with others; 3 = Raspberry; 4 = Black grape (seeds); 5 = Black grape (peel) with others and grapevine; 6 = Artichoke; 7 = Turmeric; 8 = Curry; 9 = Coffee; 10 = Pepper; 11 = Tea; 12 = Juice; 13 = Wine; 14 = Beer; 15 = Sparkling wine; 16 = Chocolate.

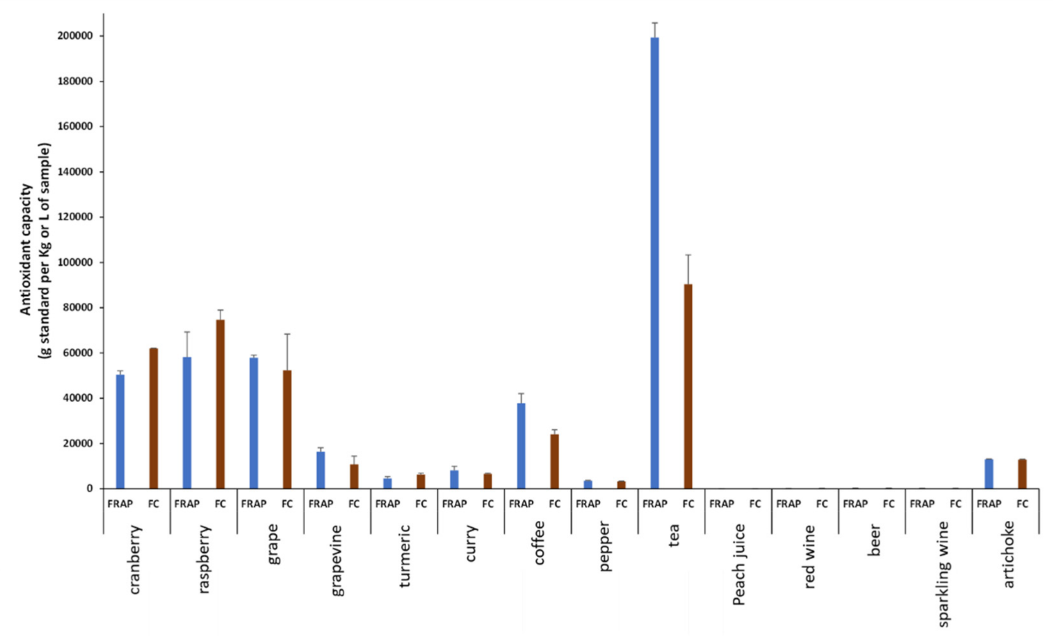

Figure S3. Determination of antioxidant capacity by FC and FRAP indexes as acid gallic or Trolox equivalents, respectively, on different samples classes. Error bars indicate the standard deviation from 3 independent replicates.

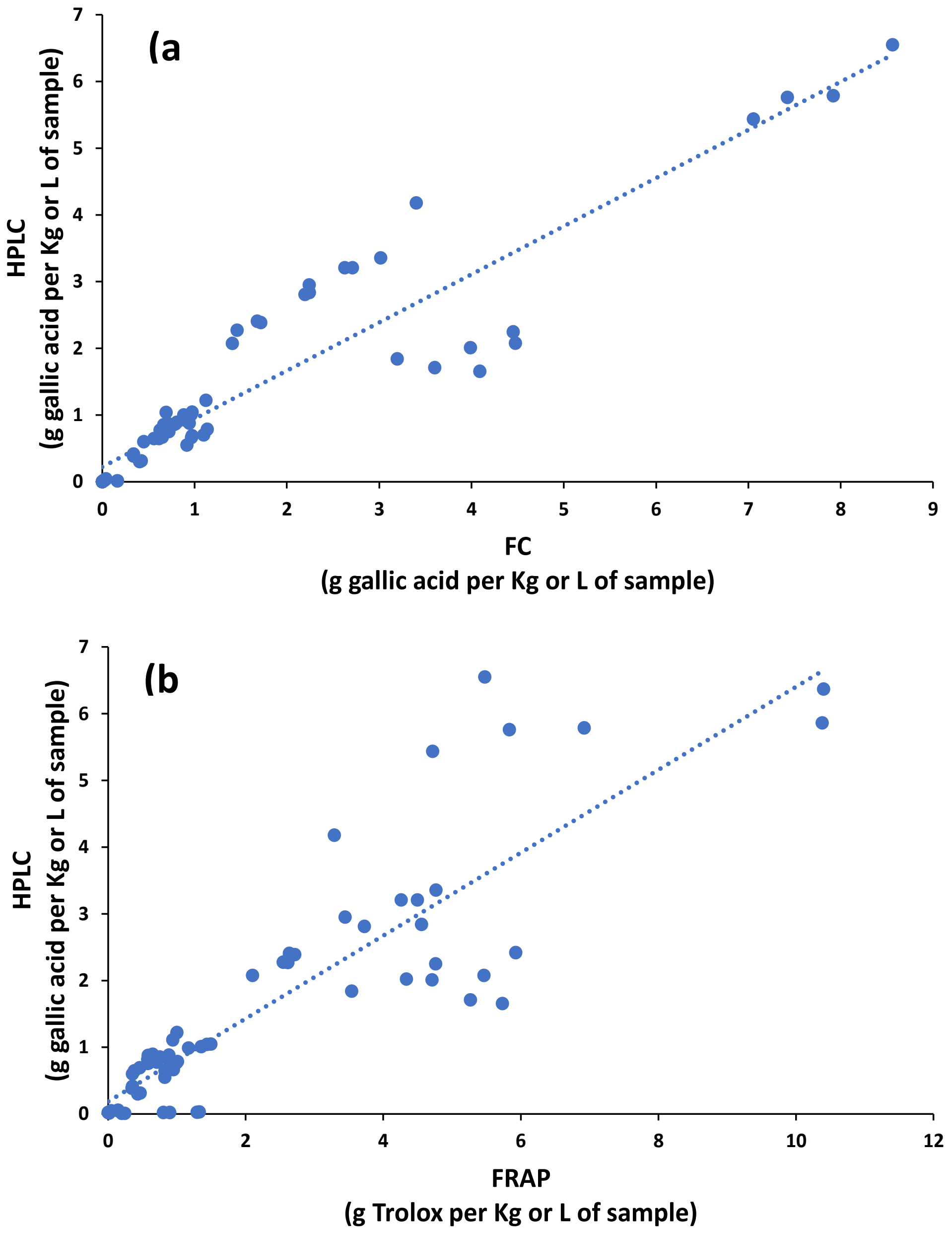

Figure S4. Correlation studies: a) FRAP versus HPLC-UV; b) FC versus HPLC-UV.

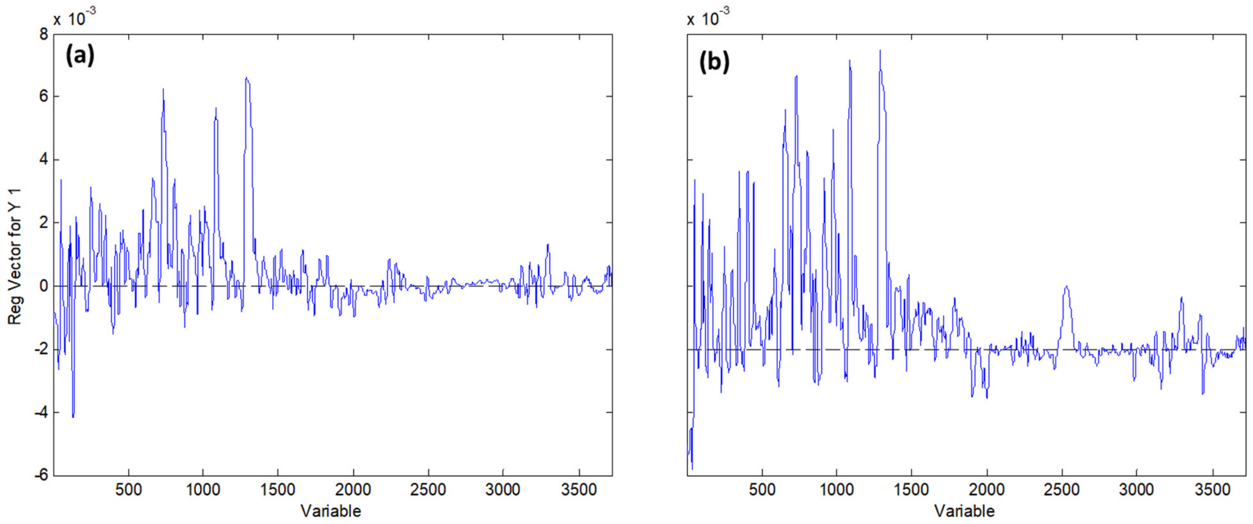

Figure S5. Regression vector for the prediction of the antioxidant index vs variables (chromatographic range): a) FC; b) FRAP.
